# The Economic Impact of Carbapenem Resistant-Non Lactose Fermenter and *Enterobacteriaceae* Infections on Hospital Costs in Dr. Soetomo General Academic Hospital Surabaya, Indonesia

**DOI:** 10.3390/antibiotics11050694

**Published:** 2022-05-20

**Authors:** Yasmeen Lashari, Maftuchah Rochmanti, Abdul Khairul Rizki Purba, Hari Basuki Notobroto, Rosantia Sarassari, Kuntaman Kuntaman

**Affiliations:** 1 Doctoral Program, Faculty of Medicine, Universitas Airlangga, Surabaya 60286, Indonesia; dr.yasmeenlashari@gmail.com; 2 Division of Pharmacology, Department of Anatomy, Histology and Pharmacology, Faculty of Medicine, Universitas Airlangga, Surabaya 60286, Indonesia; maftuchah-r@fk.unair.ac.id (M.R.); khairul_purba@fk.unair.ac.id (A.K.R.P.); 3 Department of Biostatistics and Population, Faculty of Public Health, Universitas Airlangga, Surabaya 60286, Indonesia; haribasuki.n@fkm.unair.ac.id; 4 Clinical Microbiology Specialist Program, Faculty of Medicine, Universitas Airlangga, Surabaya 60286, Indonesia; santisarassari@yahoo.com; 5 Department of Medical Microbiology, Faculty of Medicine, Universitas Airlangga, Surabaya 60286, Indonesia; 6 Dr. Soetomo General Academic Hospital, Surabaya 60286, Indonesia

**Keywords:** carbapenem-resistance, *Acinetobacter baumanni*, *Pseudomonas aeruginosa*, hospital costs, infectious diseases, Indonesia

## Abstract

Background: Carbapenem resistant-non lactose fermenter (CR-NLF) and Carbapenem resistant-*Enterobacteriaceae* (CR-E) bacterial infections are likely to be a global threat to people’s health. However, studies on the economic impacts according to the hospital setting are very scarce. The study aimed to explore the impact of CR-NLF (*Acinetobacter baumannii* = CRAB) & *Pseudomonas aeruginosa =* CRPA) and CR-E (*Escherichia coli =* CREC) & *Klebsiella pneumoniae* = CRKP) infections on hospital costs from a payer perspective among patients admitted to Dr.Soetomo Hospital, Surabaya, Indonesia. Methods: In the retrospective case-control study, medical records of all included patients hospitalized during 2018–2021 were reviewed for CRAB, CRPA, CREC, CRKP, and carbapenem sensitive (CSAB, CSPA, CSEC, CSKP) were collected. We retrieved the data of age, gender, clinical specimen, dates of admission, and discharge status. The outcomes of interest were hospital length of stay and hospitalization cost. Results: The cost for CR-NLFs infections was higher than carbapenem sensitive, $3026.24 versus $1299.28 (*p* < 0.05). There was no significant difference between CR-E against carbapenem sensitive. It showed that the highest impact of the cost was CRAB, followed by CRPA, CRKP, and CREC. The bed, antibiotics, pharmacy, and diagnostic costs of CR-NLFIs were significantly higher than CR-E. Conclusion: This study showed that the hospital cost and expenditure of CR-NLFs per patient were higher than CS. The hospital cost per patient for CR-NLF was higher than CR-E.

## 1. Introduction

Carbapenem resistant-*Enterobacteriaceae* (CR-E) and carbapenem resistant *Acinetobacter baumannii* (CRAB) are urgent threats [1] that cause severe healthcare-associated infections (HAIs) especially in critically ill and immunocompromised subjects [2], associated with significant morbidity, mortality [3], and increased healthcare costs [4]. *Acinetobacter baumannii* with minor frequency put a noteworthy burden on hospitals. The annual cost of CR-E is higher than various acute and chronic diseases [5,6]. Carbapenem resistant *Pseudomonas aeruginosa* (CRPA) patients’ death risk is more than carbapenem sensitive *Pseudomonas aeruginosa* (CSPA) [7], as *Acinetobacter baumannii*, *Pseudomonas aeruginosa*, *Klebsiella pneumoniae* and *Escherichia coli* which included in WHO critical priority pathogens resistant to carbapenems and correlated to clinical and economic burden [8]. An estimated 700 thousand people die each year due to drug-resistant infections [9], more than 70% of mortality cases are due to difficulty in CR-E treatment [10], and CRAB increases total medical cost 1.5 times more and put a heavy financial burden on hospitals [11]. If not taken seriously, more than 10 million deaths annually can occur by 2050, and a reduction to 3.8% in the annual gross domestic product (GDP) and an increase in vulnerable to poor people living in low- and middle-income countries (LMICs) [12]. Coronavirus disease 2019 (COVID-19) emerged as an alarming situation of antimicrobial resistance (AMR) [13]. During the pandemic, it is evident that the management of coronavirus infections, especially in critically ill patients, is handled with antibiotics. Therefore, the concern about antibiotic resistance arises [14]. The screening for COVID-19 and precautionary use of personnel protective equipment (PPE) put an extra financial burden on patients and hospitals [15]. COVID-19 executes a considerable financial burden on economic stability, healthcare systems, and insurance reimbursement [16]. A study from China on the economic evaluation of infection by carbapenem resistant Gram-negative bacteria was higher than carbapenem sensitive Gram-negative bacteria and demonstrated a higher impact, especially in patients of *Acinetobacter* *baumannii*, *Pseudomonas aeruginosa*, and *Klebsiella pneumoniae* [17]. Therefore, in 2015, in the World Health Assembly (WHA) directive, the World Health Organization (WHO) intended to step up the world global action plan on AMR. The goals were prevention of infectious diseases, ensuring effective treatment continuity, safe medicines, and awareness about AMR [18]. In 2017 after adopting a global action plan on AMR, the WHA members were enthusiastic about having a national action plan on AMR [19]. Indonesia, with 273.4 million people, is the fourth most populated country worldwide [20], where 70% of pharmacies sell antibiotics without prescription [21]; and the incidence of Gram-negative infections (GNIs) is on the rise [22]. *Klebsiella pneumoniae* and *Escherichia coli* develop resistance against generally available antibiotics [23]. Kuntaman et al. reported that there were 22 strains of carbapenemase-producing Gram-negative bacteria resistant to imipenem and meropenem [24]. Gram-negative isolates were more common than Gram-positive isolates, and *Acinetobacter baumannii,* *Klebsiella pneumoniae*, and *Escherichia coli* were more frequent than other Gram-negative bacteria (GNB). The sensitivity to carbapenem ranged between 20.0% to 66.0% in *Acinetobacter baumannii* [25]. With the help of the WHO, the Ministry of Health (MOH) Indonesia launched an antimicrobial stewardship program and developed committees at the national and hospital level to prevent excessive use of antibiotics [26]. In 2019, several meetings discussed the activities required to face future challenges and endorsed the national action plan to control AMR [27]. Carbapenems continue to play a critical role as some of the agents of last resort for the treatment of antibiotic-resistant in GNIs [28]. Meanwhile, hospital length of stay (LoS), in-hospital mortality rate, and hospitalization costs were high in carbapenem-resistant *Klebsiella pneumoniae* CRKP, CRPA, and CRAB versus carbapenem-sensitive (CS) [29,30]. The costs of SSI (surgical site infection) might increase to US$22,130 per patient due to readmission, prolonged LoS, and medicines [31]. However, the microbiological culture analysis-based treatment can increase life expectancy and save costs, especially in intensive care units (ICUs) during hospitalization [32]. Carbapenem resistance definition is by way of the minimum inhibitory concentration (MIC) value used to categorize an organism as sensitive (S), intermediate (I), or resistant (R). The MIC breakpoint for *Enterobacterales* resistance to doripenem is ≥4, ertapenem ≥ 2, imipenem ≥ 4, and meropenem ≥ 4 μg/mL. *Pseudomonas aeruginosa* resistance to doripenem is ≥8, imipenem ≥ 8, and meropenem ≥ 8 μg/mL; and for *Acinetobacter spp* to doripenem is ≥8, imipenem ≥ 8 and meropenem ≥ 8 [33]. The national data on AMR and its impact on hospitalization costs are not available. The imipenem-resistance rate in Indonesia is the highest among Asian countries [34]. However, very little was known about hospital outcomes and costs associated with HAIs, particularly CR-NLFs, Carbapenem sensitive-non lactose fermenters (CS-NLFs), and CR-E, Carbapenem sensitive-*Enterobacteriaceae* (CS-E). Therefore, this study was aimed to estimate and explore the impact of the carbapenem-resistant and sensitive non-lactose fermenter and *Enterobacteriaceae* infections on LoS and costs; and to estimate the impact of the COVID-19 pandemic on LoS and hospitalization costs related to carbapenem-resistant and sensitive non-lactose fermenter & *Enterobacteriaceae* infections among patients admitted in the Dr. Soetomo Hospital, Surabaya, Indonesia.

## 2. Results

The study started from January 2021 until August 2021. The data were extracted from the electronic medical record from March 2018 until February 2021. The study included 810 cases, and 270 (33.3%) were found carbapenem-resistant. There were 431 females, and 31.1% were resistant, while among males, 35.9% were resistant. This difference between two genders was insignificant (35.9% vs. 31.1%; *p* = 0.149). The median age was 55.0 (44.0–64.0) for sensitive cases and 53.0 (42.0–61.0) years for resistant cases, and the difference was insignificant, with a *p*-value of 0.070. Most common specimen were urine *n* = 567, 70% followed by blood *n* = 188, 23%, pleural fluid *n* = 22, 2.7% and CSF (cerebrospinal fluid) *n* = 15, 1.9%, and peritoneal *n* = 10, 1.2% and then few cases of others. Among clinical specimens, carbapenem-resistant cases were more frequent in CSF (46.7%), followed by blood (37.8%), urine (32.8%), peritoneal fluid (30.0%), and pleural fluid (13.6%). However, the frequency of carbapenem-resistant cases was not significantly different across different specimens, with a *p*-value of 0.054. The distribution of carbapenem-resistant cases was also not significantly different between organisms and their subtypes (Table 1).

Both carbapenem-resistant and sensitive cases were categorized into four groups each, i.e., carbapenem-resistant EC + KP, carbapenem-resistant PA + AB, carbapenem-sensitive EC + KP, and carbapenem-sensitive PA + AB. All were found significantly different among four groups with *p* < 0.05. The carbapenem-resistant group with PA + AB had a significantly higher median cost of $3026.2 as compared to the carbapenem-sensitive group and had a significantly higher median cost as compared to the carbapenem-resistant group EC + KP with *p*-values < 0.001. When hospital cost was compared between two groups, the pre-pandemic (March 2018–February 2020) (*n* = 700) and pandemic (March 2020–February) (*n* = 110), irrespective of carbapenem status, the pandemic median cost of PA + AB and EC + KP was higher than the pre-pandemic but we did not find any significant difference in the cost (Table 2).

The median LoS was 15.0 (IQR, 9.0–26.0) days for carbapenem-resistant cases, which was significantly more prolonged than 11.0 (IQR, 7.0–20.0) days for carbapenem-sensitive cases (*p* < 0.001). The carbapenem-resistant cases’ median bed cost was $249.1, for antibiotics $45.4, for pharmacy $601.5, for diagnostic $597.4, and $1536.4 was the total hospital cost and all were significantly higher as compared to sensitive cases with *p*-values < 0.05. The other costs were insignificantly different between the two groups, with a *p*-value of 0.933. (Table 3).

Both carbapenem-resistant and sensitive cases were categorized into two groups each, i.e., carbapenem-resistant EC + KP, carbapenem-resistant PA + AB, carbapenem-sensitive EC + KP, and carbapenem-sensitive PA + AB. Median LoS and various costs were compared, and all of them were found to be significantly different between the four groups with *p*-values < 0.05. The carbapenem-resistant PA + AB had significantly higher median costs of bed $827.1, antibiotics $77.6, pharmacy $1147.3, diagnostics $783.8 and total treatment cost $3026.2 as compared to carbapenem-resistant EC + KP, carbapenem-sensitive PA + AB and carbapenem-sensitive EC + KP with *p*-values < 0.05. The cost of antibiotics for carbapenem-resistant EC + KP was also significantly higher than for carbapenem-sensitive EC + KP, with a median cost of $32.3 vs. $16.7 (Table 4).

The median costs of beds, antibiotics, pharmacy, diagnostics, and total hospital costs increased with the increase in LoS. The median total cost for a stay ≤5 days was $580.4, which reached $799.8 for a stay of 6–10 days, $1284.7 for 11–15 days, and 2802.7 for a stay of more than 15 days (Table 5)

When the hospital cost was compared between two groups, i.e., COVID-19 pre-pandemic cases and pandemic cases, irrespective of carbapenem status, the median cost of antibiotics was $13.3 in pandemic cases, which was significantly lower compared to $26.8 in pre-pandemic cases (*p* = < 0.001). However, the median cost of diagnostics of $599.4 in pandemic cases was significantly higher than $479.6 in pre-pandemic cases (*p* = 0.034). Similarly, the median cost of other costs was $2.7 in pandemic cases, which was significantly higher than $2.4 in pre-pandemic cases (*p* = 0.004). Among carbapenem-sensitive, the median cost of antibiotics was significantly lower in pandemic cases than in pre-pandemic cases (*p* = < 0.001). Among carbapenem-resistant, the median costs of diagnostics (*p* = 0.019) and others (*p* = 0.048) were significantly higher in pandemic cases than the pre-pandemic cases. (Table 6).

## 3. Discussion

Carbapenem was recognized two decades ago as a very effective broad-spectrum antibiotic with fewer side effects. It is commonly used for multidrug-resistant strains treatment as the last choice [35,36]. In the β-lactam family, the carbapenems are important in anti-bacterial and distinctively steady in most β-lactamases [37]. The impact of carbapenem-resistance (CR) has been examined earlier in GNI patients. More studies are needed to independently determine the economic cost of NLF (*Acinetobacter bamuanai* and *Pseudomonas aeruginosa*) and CR-E (*Escherichia coli* and *Klebsiella pneumoniae*) to show a more inclusive picture of infection cost. As compared to the west, Asia experienced more burden of Gram negative-resistant [38]. At the beginning of the 21st century, *Enterobacteriaceae* was reportedly identified in the USA and later on globally [39]. According to US Premier Healthcare Database (2014–2019), an analysis of CR demonstrated an increased cost of hospitalization than CS [40] and is associated with increased mortality, costs, and prolonged stay [41]. According to our knowledge, this is the first study conducted to evaluate outcome and hospital cost statistically to compare the financial burden of carbapenem-resistant versus carbapenem-sensitive GNIs in hospitalized patients in Indonesia. Therefore, this study aimed to estimate and explore the impact of carbapenem-resistant and sensitive *Enterobacteriaceae* and non-lactose fermenter infections on hospital outcomes and costs among patients admitted to the Dr. Soetomo Hospital Surabaya Indonesia. The present study showed that LoS and hospitalization costs were high in carbapenem-resistant versus sensitive. And CR-NLF infections LoS and hospitalization costs were higher than the CR-E. These results are in agreement with the findings reported in other studies. In a recently published systematic review and meta-analysis, Avendano et al. concluded that carbapenem-resistant GNIs in high-risk patients were associated with increased mortality, emphasizing antimicrobial stewardship and infection control in hospitals [42]. Likewise, Zhen et al. reported that CRKP, CRPA, and CRAB were associated with significantly higher hospital costs and prolonged hospital stay [10]. We found that some patients infected with *Pseudomonas aeruginosa* or *Acinetobacter baumannii* as a group of non-lactose fermenters were primarily a late infection or patients with chronic diseases and more therapeutic medicine. This also showed in this study that LoS patients infected with CRAB/CRPA are longer than the sensitive group. In the same way, Priyendu et al. found that the costs of overall treatment and hospital LoS were higher in the CRKP group than in the CSKP group. The authors concluded that carbapenem resistance could lead to a greater burden of treatment costs for patients [43]. Lemos et al. also calculated higher treatment costs in the CRAB group as compared to the CSAB group. However, LoS in the ICU and hospital mortality were not significantly different between CR and CS groups [44]. In the study, carbapenem-sensitive cases were more frequent in urine, followed by blood, pleural fluid, CSF, and peritoneal fluid. However, the frequency of carbapenem-resistant cases was not significantly different across different specimens. Luke et al. reported that the most frequent infections were respiratory infections, followed by urinary tract, systemic, and skin infections in overall CRKP and CSKP infections [45]. Among demographic characteristics evaluated in the study, age was not significantly different between the two groups by carbapenem status. However, gender male was significantly associated with carbapenem resistance. Whereas Zhen et al. found significant age and gender differences between CRAB versus CSAB and CRPA versus CSPA; these differences were not significant between CRKP versus CSKP [17]. When the costs were compared between cases before the COVID-19 pandemic and during the pandemic, it was observed that the antibiotic cost was lower during the pandemic than before the pandemic. Likewise, in Scotland, a study showed the same trends of decreased antibiotic prescribing during 2020 compared to pre-pandemic [46]. Comparing the bed, pharmacy, and total costs was lower, similarly Best et al. found that the revenue of American hospitals drops $16.3 to $17.7 billion due to canceled hospital services because of the COVID-19 pandemic [47]. Our study has several notable strengths. The present study is the first in Indonesia to examine the economic impacts of carbapenem resistance among patients with NLFs and *Enterobacteriaceae*. Comparatively, a study done in 2020 was a retrospective study, inherently resulting in bias and confounding. PSM (propensity score matching) was conducted to balance potential confounding factors to minimize the risk of bias, but the PSM ignores unmeasured confounders that could potentially impact outcomes [17]. But our study was case-control. We used case-control in this study design. A case-control study is always retrospective because it starts with an outcome and then traces back to investigate exposure allowing for meaningful comparison of CREC with CSEC, CRKP with CSKP, CRAB with CSAB, and CRPA with CSPA. Our study strengthens the case-control match study by using this method to eliminate the effects of confounding factors. However, the main potential benefit of matching in case-control studies is a gain in efficiency [48]. Some limitations are worth noting. Our study was conducted in a tertiary referral hospital and A-type hospital. The results may not be generalizable to other medical institutions and hospitals like B, C, and D types. It is necessary to expand this study to different types of hospitals in various areas in the future and also include clinical outcomes to provide inclusive sight related to infections cost and the impact of carbapenem resistance who were infected with NLF (*Acinetobacter baumannii* and *Pseudomonas aeruginosa*) and CR-E (*Escherichia coli* and *Klebsiella pneumoniae*).

## 4. Materials and Methods

### 4.1. Design and Population Study

We performed a retrospective case-control study with cost analysis using a payer perspective in a tertiary referral hospital Dr. Soetomo Hospital, Surabaya, Indonesia, with 1514 beds and 26 departments serving eastern Indonesia. The ethical committee of Dr. Soetomo Hospital approved this study (No. 0188/KEPK/IV/2021) dated 29th April 2021. Patients were identified from the records of the clinical microbiology laboratory patients. Data on medical/pharmaceutical/operating costs were obtained from electronic medical records, accounting department, and finance department records from March 2018 to February 2021.

We expected that NLF and *Enterobacteriaceae* infections due to resistance compared with sensitive bacteria were the independent exposure of interest. We estimated their impact on excess LOS and excess hospital charges.

### 4.2. Criteria for Selecting Patients

The criteria included those aged 18 years and above, including males and females, admitted to surgical, medical, and ICU wards. Patients with clinical samples positive for CRAB, CRPA, carbapenem-resistant *Escherichia coli* (CREC), and CRKP infection were included in the case group. We collected the control group from all carbapene-sensitive strains of patients, namely CSAB, CSPA, carbapenem sensitive *Escherichia coli* (CSEC), and CSKP. Patients with COVID-19 were excluded.

### 4.3. Data Collection

The data source was the patient’s medical records. We included patient demographics such as age, sex and clinical specimen, hospital events admitting service, surgical and medical services, and hospital and ICU admission/discharge dates.

### 4.4. Cost Calculation

The retrospective data included age, sex, and hospital events (admitting service, surgical & medical services, dates of admission, discharge from hospital, and ICU). The study outcomes included hospital LoS and hospitalization costs such as bed charges, antibiotics, pharmacy, laboratory, radiology procedures, other medical facilities costs, and total costs covered by health insurance. Bed costs included hospital administration fees, daily room service, nursing and medical staff care, and technicians’ services. We referred to the Pharmacy department to extract the antibiotics costs. Pharmacy costs covered many expenditures, including drugs, fluids, blood products for transfusion, disposable devices, mechanical ventilators, oxygen therapy, and pharmacy services. The service charge for Physiotherapists and consultancy fees were under bed service costs. The costs of other medical facilities were including of administration, ticket costs, ambulance, patient transfer, and others. The currency exchange rate 2021 (US $1 = 13,308.15 IDR) was used, values of the economic cost convert Indonesian Rupiahs (IDR) into US Dollars (US $) [49].

### 4.5. Outcomes

The primary outcomes were to estimate extended LoS due to CRAB, CRPA, CREC, and CRKP compared with sensitivity CSAB, CSPA, CSEC, and CSKP bacteria from the payer perspective and compare the differences in the results between cases and control.

### 4.6. Statistical Analysis

The data analysis was conducted using the IBM Statistics SPSS version 20.0. Gender, organism, specimen, and other drug sensitivity were shown with frequency and percentages. A comparison between two groups by carbapenem resistance was performed using Chi-square and likelihood ratio test. We used the median (IQR) for groups by carbapenem resistance to describe the status data of age, length of stay, and costs for different factors. The analysis between groups used the Mann–Whitney U test. The comparison among four groups by carbapenem status, organism type, and LoS using Kruskal–Wallis followed by Mann–Whitney for pairwise comparison. Comparison of the cost between pre-pandemic and pandemic period for all cases, between carbapenem-resistant and sensitive cases, used the Mann–Whitney U test. *p*-value < 0.05 was considered significant.

## 5. Conclusions

The present study concluded that length of stay and hospitalization cost was high in carbapenem-resistant versus sensitive and higher in CR-NLF than in CR-E infections. Furthermore, the costs of diagnostics and others were higher in pandemic cases than in pre-pandemic cases, irrespective of their carbapenem status among patients admitted to the Dr. Soetomo Hospital Surabaya Indonesia.

## Figures and Tables

**Table 1:** Distribution of cases by gender, specimen, organism, and outcome as per carbapenem status.

		Carbapenem				
		Sensitive		Resistant		
		*n*	%	*n*	%	*p*-Value
Total		540	55.0	270	53.0	0.070
Sex	Female	297	68.9	134	31.1	0.149
	Male	243	64.1	136	35.9	
Specimen	Urine	381	67.2	186	32.8	0.054
	Blood	117	62.2	71	37.8	
	Pleural Fluid	19	86.4	3	13.6	
	CSF	8	53.3	7	46.7	
	Peritoneal	7	70.0	3	30.0	
	Pericardial	3	100.0	0	0.0	
	Others Sterile Fluid	3	100.0	0	0.0	
	Joint Fluid	2	100.0	0	0.0	
Organism	*Escherichia coli*	244	66.7	122	33.3	1.000
	*Klebsiella pneumoniae*	72	66.7	36	33.3	
	*Pseudomonas aeruginosa*	92	66.7	46	33.3	
	*Acinetobacter baumannii*	132	66.7	66	33.3	
Bacterial Organism category	EC + KP	316	66.7	158	33.3	1.000
	PA + AB	224	66.7	112	33.3	

Note: EC: Escherichia coli, KP: Klebsiella pneumoniae, AB: Acinetobacter baumannii, PA: Pseudomonas aeruginosa, statistically significant, *p* < 0.05.

**Table 2:** Comparison of various hospital median costs among four groups by carbapenem status, organism’s category, and pre-pandemic versus pandemic (in 2021 US$).

Organisms	Carbapenem		*p*-Value	Pre- Pandemic	Pandemic	*p*-Value
	Sensitive	Resistant				
PA + AB (NLF)	1299.28	3026.24	<0.001	1593.7523	1792.01	0.526
EC + KP (Enterobacteriaceae)	1061.2091	1179.31	0.457	1060.71	1333.66	0.254
AB	1266.17	3421.55	<0.001	1587.05	2139.22	0.323
PA	1368.21	2534.55	0.009	1740.31	1248.23	0.778
EC	1048.35	1102.97	0.943	1012.48	1473.28	0.083
KP	1178.18	1777.64	0.139	1301.53	1076.87	0.629

Note: NLF: non lactose fermenter, AB: *Acinetobacter baumannii*, PA: *Pseudomonas aeruginosa*, EC: *Escherichia coli*, KP: *Klebsiella pneumoniae*, pre-pandemic: March 2018–February 2020; pandemic. March 2020–February 2021, statistically significant, *p* < 0.05.

**Table 3:** Comparison length of stay and various kind of costs between carbapenem-sensitive and resistant cases (in 2021 US$).

Hospital Cost	Carbapenem		
	Sensitive (*n* = 540) Median (IQR)	Resistant (*n* = 270) Median (IQR)	*p*-Value
Bed cost	198.1 (72.1–759.5)	249.1 (80.7–1242.6)	0.024
Antibiotic cost	18.5 (6.6–51.3)	45.4 (14.7–126.3)	<0.001
Pharmacy cost	402.7 (214.6–841.7)	601.5 (314.0–1373.0)	<0.001
Diagnostic cost	447.7 (298.7–708.0)	597.4 (376.4–967.2)	<0.001
Other cost	2.4 (2.4–2.7)	2.4 (2.4–2.7)	0.933
Total cost	1150.0 (712.2–2208.2)	1536.4 (833.3–3797.7)	<0.001
LoS	11.0 (7.0–20.0)	15.0 (9.0–26.0)	<0.001

Note: Other costs = Administrative costs, ticket costs, and ambulance costs. IQR: Interquartile range, statistically significant, *p* < 0.05.

**Table 4:** Costs and length of stay for various components among four groups by carbapenem status and organism type (in 2021 US$).

Hospital Cost	Carbapenem Sensitive		Carbapenem Resistant		*p*-Value
	EC + KP (*n* = 316) Median (IQR)	PA + AB (*n* = 224) Median (IQR)	EC + KP (*n* = 158) Median (IQR)	PA + AB (*n* = 112) Median (IQR)	
Bed cost	176.1 (70.2–653.3)	218.9 (74.7–837.2)	153.2 (62.7–596.7)	827.1 (151.9–2354.1)	<0.001
Antibiotic cost	16.7 (6.6–50.6)	21.8 (6.7–51.9)	32.3 (10.7–87.3)	77.6 (27.3–249.7)	<0.001
Pharmacy cost	363.7 (208.8–738.1)	453.9 (217.1–918.2)	454.1 (268.0–830.3)	1147.3 (545.1–2331.3)	<0.001
Diagnostic cost	438.5 (291.2–645.9)	457.6 (308.7–788.5)	489.1 (324.1–743.1)	783.8 (512.1–1307.4)	<0.001
Other costs	2.4 (2.4–2.7)	2.6 (2.4–2.7)	2.7 (2.4–2.7)	2.4 (2.4–2.7)	0.047
Total cost	1061.2 (697.2–2047.2)	1299.3 (752.3–2644.6)	1179.3 (709.3–2160.2)	3026.2 (1217.8–5719.9)	<0.001
LoS	10.5 (7.0–19.0)	13.0 (8.0–21.0)	13.0 (7.0–22.0)	18.0 (11.0–31.5)	<0.001

Note: IQR: Interquartile range, statistically significant, *p* < 0.05.

**Table 5:** Comparison of various costs per patient by the length of stay (in 2021 US$).

	Length of Stay (days)				*p*-Value
Hospital Costs	≤5 (*n* = 106) Median (IQR)	6–10 (*n* = 236) Median (IQR)	11–15 (*n* = 160) Median (IQR)	>15 (*n* = 308) Median (IQR)	
Bed cost	56.2 (30.5–137.5)	90.3 (49.7–232.8)	206.3 (85.8–656.4)	796.1 (262.9–1982.2)	<0.001
Antibiotic cost	5.2 (2.0–11.2)	16.3 (6.3–31.6)	25.2 (9.2–51.8)	74.9 (28.8–168.3)	<0.001
Pharmacy cost	179.6 (86.8–284.8)	305.4 (183.6–455.5)	444.2 (283.0–694.4)	1011.3 (607.2–2082.6)	<0.001
Diagnostic cost	290.4 (214.9–411.9)	365.2 (267.5–510.8)	492.1 (333.6–655.5)	852.2 (562.5–1314.7)	<0.001
Other costs	2.4 (2.4–2.7)	2.4 (2.4–2.7)	2.4 (2.4–2.7)	2.7 (2.4–3.1)	0.011
Total cost	580.4 (424.1–833.9)	799.8 (618.5–1182.8)	1284.7 (892.3–1888.7)	2802.7 (1697.4–5200.8)	<0.001

Note: IQR: Interquartile range, statistically significant, *p* < 0.05.

**Table 6:** Comparison of various costs by Carbapenem-resistance, susceptible, and duration of pre-pandemic and pandemic (COVID-19) (in 2021 US$, World Bank 2021).

Hospital Costs	Carbapenem Sensitive			Carbapenem Resistant			Overall		
	Pre-Pandemic (*n* = 464)	Pandemic (*n* = 76)	*p*-Value	Pre-Pandemic (*n* = 236)	Pandemic (*n* = 34)	*p*-Value	Pre-Pandemic (*n* = 700)	Pandemic (*n* = 110)	*p*-Value
Bed cost	177.7 (67.9–785.3)	271.6 (114.1–614.0)	0.300	227.5 (75.3–1253.9)	419.6 (156.2–1052.5)	0.122	195.4 (71.0–932.6)	288.2 (130.3–654.5)	0.090
Antibiotic cost	20.5 (7.5–56.0)	8.9 (3.9–33.9)	<0.001	46.8 (15.5–131.3)	33.7 (8.8–88.5)	0.221	26.8 (8.5–83.1)	13.3 (5.2–47.1)	<0.001
Pharmacy cost	398.3 (209.8–855.0)	415.7 (231.4–778.9)	0.925	585.5 (304.5–1441.1)	736.4 (408.9–1240.5)	0.612	459.3 (236.0–993.6)	463.0 (251.9–850.8)	0.955
Diagnostic cost	437.2 (298.7–698.1)	541.0 (305.0–778.0)	0.279	567.3 (366.5–958.7)	756.2 (543.9–1067.3)	0.019	479.6 (311.3–776.6)	599.4 (375.4–849.6)	0.034
Other cost	2.4 (2.4–2.7)	2.7 (2.4–2.7)	0.034	2.4 (2.4–2.7)	2.7 (2.4–2.7)	0.048	2.4 (2.4–2.7)	2.7 (2.4–2.7)	0.004
Total cost	1119.8 (700.8–2262.8)	1318.4 (800.0–2059.6)	0.613	1462.1 (800.2–3877.8)	1943.3 (1215.6–3517.3)	0.221	1237.3 (732.7 –2760.4)	1535.0 (869.2–2281.2)	0.313

Note: IQR: Interquartile range, Pre-pandemic: March 2018–February 2020; Pandemic. March 2020–February 2021, statistically significant, *p* < 0.05.

## Data Availability

The data will be provided upon worthy reasonable request from the corresponding author.

## Abbreviations

AB = *Acinetobacter baumannii*; AMR = Anti-microbial resistance; CR = Carbapenem-resistant; CRAB = Carbapenem resistant *Acinetobacter baumannii*; CR-E = Carbapenem resistant-*Enterobacteriaceae*; CREC = Carbapenem resistant *Escherichia coli*; CR-GNB = carbapenem resistant-Gram negative bacteria; CRKP = Carbapenem resistant *Klebsiella pneumoniae*; CR-NLF = Carbapenem resistant-non lactose fermenter; CRPA = Carbapenem resistant *Pseudomonas aeruginosa*; CS = Carbapenem-sensitive; CSAB = Carbapenem sensitive *Acinetobacter baumannii*; CS-E = Carbapenem sensitive-*Enterobacteriaceae*; CSEC = Carbapenem sensitive *Escherichia coli*; CSF = Cerebrospinal fluid; CS-GNB = Carbapenem sensitive-Gram negative bacteria; CSKP = Carbapenem sensitive *Klebsiella pneumoniae*; CSPA = Carbapenem sensitive *Pseudomonas aeruginosa*; Carbapenem sensitive-non lactose fermenter (CS-NLF); COVID-19 = Coronavirus disease 2019; EC = *Escherichia coli*; GDP = Gross domestic product; GNB = Gram-negative bacteria; GNI = Gram-negative infection; HAIs = Healthcare-associated infections; ICUs = Intensive care units; IDR = Indonesian Rupiahs; IQR = Interquartile range; KP = *Klebsiella pneumoniae*; LMICs = Low- and middle-income countries; LoS = Length of stay; NLF = Nonlactose fermenter; PA = *Pseudomonas aeruginosa*; PPE = Personnel protective equipment; SSI = Surgical site infection; WHA = World Health Assembly; WHO = World Health Organization.
